# *Bordetella pertussis*: an underreported pathogen in pediatric respiratory infections, a prospective cohort study

**DOI:** 10.1186/1471-2334-14-526

**Published:** 2014-09-30

**Authors:** Gertrude van den Brink, Jérôme O Wishaupt, Jacob C Douma, Nico G Hartwig, Florens GA Versteegh

**Affiliations:** Department of Pediatrics, ErasmusMC-Sophia, Wytemaweg 80 3015 CN, Rotterdam, The Netherlands; Department of Pediatrics, Reinier de Graaf Hospital, Delft, The Netherlands; Centre for Crop System Analysis, Wageningen University and Research Centre, Wageningen, The Netherlands; Department of Pediatric Infectious Diseases and Immunology, ErasmusMC–Sophia, Rotterdam, The Netherlands; Sint Franciscus Gasthuis, Rotterdam, The Netherlands; Department of Pediatrics, Groene hart Ziekenhuis Gouda, Gouda, The Netherlands; Department of Pediatrics, Ghent University Hospital, Gent, Belgium

**Keywords:** *Bordetella pertussis*, Whooping cough, Respiratory tract infections, Polymerase chain reaction, Child

## Abstract

**Background:**

The incidence of pertussis has been increasing worldwide. In the Netherlands, the seroprevalence has risen higher than the reported cases, suggesting that laboratory tests for pertussis are considered infrequently and that even more pertussis cases are missed. The objective of our study was to determine the frequency of pertussis in clinically unsuspect cases compared to suspect cases with the intention of finding clinical predictors.

**Methods:**

The present prospective cohort study was part of a controlled clinical trial evaluating the impact of molecular diagnostics on clinical decision making in pediatric respiratory infections, performed during 2 winter seasons. For this study, in the first season pertussis was only tested in case of clinical suspicion, in the second season, pertussis was also tested without clinical suspicion. Multivariate and univariate analysis were performed using SPSS 18 and Statistical software ‘R’.

**Results:**

In the two seasons respectively 22/209 (10,5%) and 49/373 (13,1%) cases were clinically suspected of pertussis. *Bordetella pertussis* was detected by real time RT-PCR in respectively 2/22 (9,1%) and 7/49 (14,3%) cases. In the second season an additional 7 cases of pertussis were found in clinically unsuspected cases (7/257 = 2,7%). These additional cases didn’t differ in clinical presentation from children without a positive test for pertussis with respect to respiratory symptoms.

**Conclusions:**

Pertussis in children sometimes mimics viral respiratory tract infections. If pertussis diagnostics are based on clinical suspicion alone, about 1 in 5 cases (19%) is missed. Despite widely accepted clinical criteria, paroxysmal cough is not a good predictor of pertussis. To prevent spreading, physicians should include *B. pertussis* in routine diagnostics in respiratory tract infections.

**Electronic supplementary material:**

The online version of this article (doi:10.1186/1471-2334-14-526) contains supplementary material, which is available to authorized users.

## Background

Currently, an increase in reported cases of pertussis is noted in many countries, even in countries with high vaccination coverage [1, 2]. However, a higher rise in seroprevalence is observed in relation to reported cases [3]. From this study one may conclude that cases of pertussis are missed. A possible explanation for this is the wide clinical spectrum of pertussis, ranging from a classical presentation with severe disease and paroxysmal cough to mild disease with only rhinitis. Life threatening disease with apneas is usually restricted to young infants that have not been (fully) immunized. The classic presentation of pertussis is well-known, but is observed less often since start of immunization. Not only immunization, but also previous infection may lead to atypical (mild) pertussis disease which is often not recognized [2, 4, 5]. These atypical cases are held responsible for ongoing transmission within the population [4, 6].

Respiratory (co-) infections may complicate a correct clinical diagnosis of *B. pertussis* infection. Several studies show that clinical presentation of pertussis is indistinguishable from viral respiratory infections and that co-infection with pertussis exists [4, 7–9]. Considering the heterogenic clinical picture of *B. pertussis* infections, the diagnosis cannot solely be made on clinical criteria. Despite this, not all countries apply laboratory tests but rely on WHO case definitions of pertussis [10]. To control the spread of pertussis within the general population more frequent laboratory confirmation should be advocated [7, 11].

Since pertussis may mimic a viral respiratory infection and present without classic symptoms, cases of pertussis are probably not recognized. The frequency of missed diagnosis is not known. Therefore, we conducted a study during two winter seasons in pediatric patients presenting with an acute respiratory tract infection (ARI). We assessed the frequency of pertussis in clinical suspected and unsuspected cases. Our primary goal was to determine the frequency of *B. pertussis* cases in the group of unsuspected children in relation to the group of clinically suspected children and to determine clinical predictors of *B. pertussis* infection in young children.

## Methods

### Study design

This prospective cohort study originated in the EVIDENCE-trial (Evaluation of Viral Diagnostics on Respiratory Infections in Children) which was designed as a multicenter, controlled, clinical trial to evaluate the impact of real-time reversed transcriptase polymerase chain reaction (RT-PCR) diagnostics in pediatric patients with ARI. ARI was defined as a new episode of respiratory symptoms of the upper and/or lower airways. The study protocol and definitions has been described before and will be summarized below [12]. The trial was conducted during 2 consecutive winter seasons (November 2007-May 2009) at the Reinier de Graaf Hospital, Delft, which was joined in the second season by the Groene Hart Ziekenhuis, Gouda, the Netherlands. Both serve as university teaching hospitals.

For this prospective cohort study of pediatric pertussis infections, specimens for pathogen diagnosis (mostly nasal wash specimens/nasopharyngeal aspirates (NWSs) or sometimes throat swabs) were collected from all children < 12 years of age with suspected ARIs who were referred to the pediatrician. All patients, or their legal representatives, gave informed consent. Clinical management was based on pediatric history and physical examination. The pediatrician decided whether to perform pertussis diagnostics on the basis of the WHO definition of pertussis. However, due to some subjective criteria in this definition, the interpretation of the criteria might be different between doctors. Therefore, strict indications for performing pertussis diagnostics were not defined in this study. The method used for detection of *B. pertussis* in these specimens was an RT-PCR targeting IS481.

**Figure 1:**
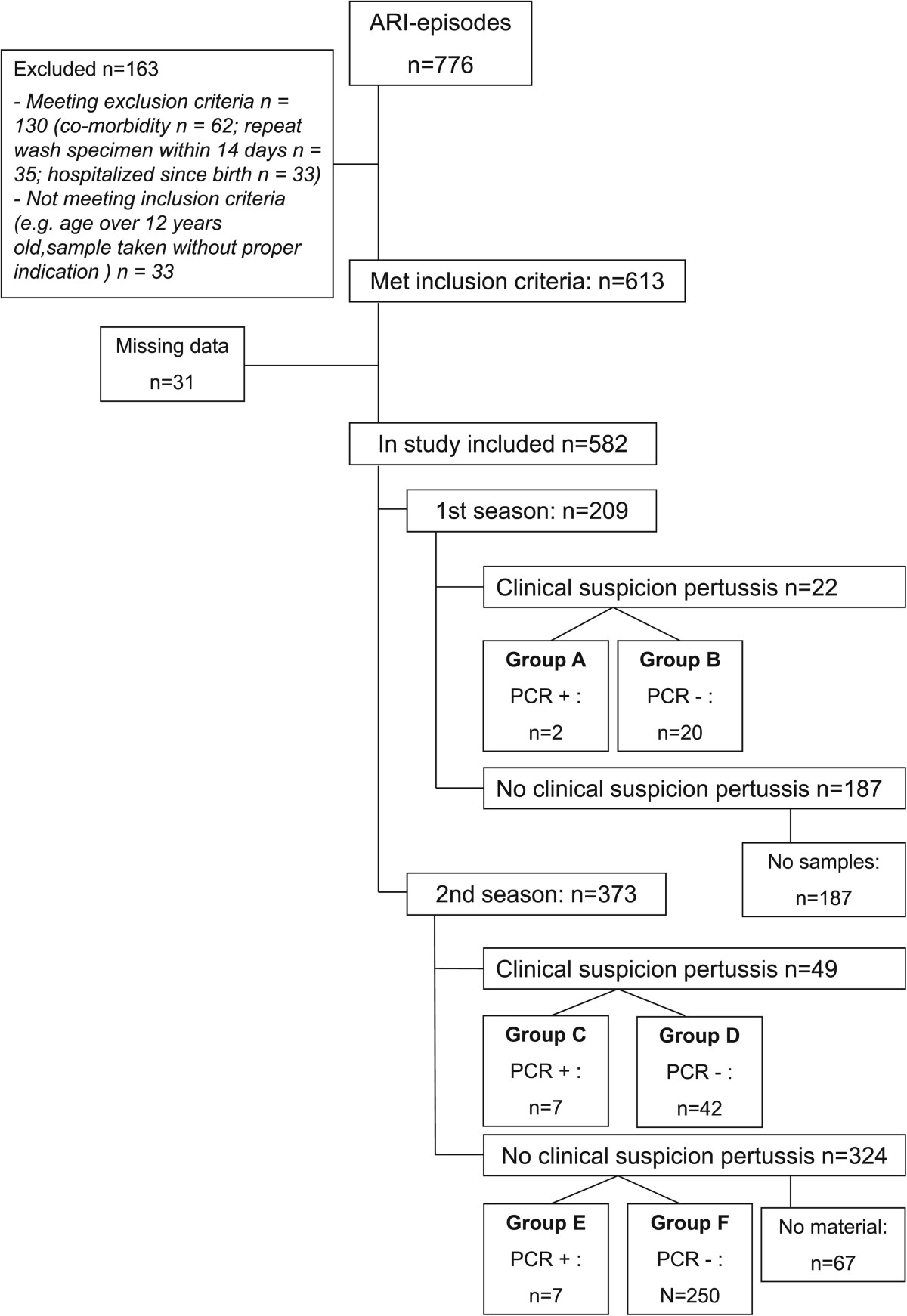
**Flowchart of patient enrollment.**

In the first winter season laboratory confirmation of *B. pertussis* infection was only performed in case of clinical suspicion of pertussis. In the second season laboratory testing was performed in all cases with or without clinical suspicion. In cases without clinical suspicion diagnostics was performed retrospectively on NSWs that were still available*.* Unfortunately, it was not possible to perform retrospective analysis on the samples of unsuspicious cases in the first winter season because those samples were not preserved (see Figure 1). Four groups were formed: 1.Clinical suspicion RT-PCR pertussis positive (Figure 1, group A and C); 2. Clinical suspicion RT-PCR pertussis negative (Figure 1, group B and D); 3. Non suspicion RT-PCR pertussis positive (Figure 1, group E) and 4. Non suspicion RT-PCR pertussis negative (Figure 1, group F).

Details about in- and exclusion criteria, definitions of upper (URTI) en lower respiratory tract infections (LRTI), data collection and sample processing have been published before [12]. Clinical data were prospectively collected for each patient by using a case report form by the clinician and missing data and laboratory parameters were retrieved from the electronical medical and hospital records. Variables used for analysis include: age, gender, fever, paroxysmal cough, rhinorrhea, wheezing, apneas, nasogastric feeding, days of illness, disease severity score, hospitalization and days in hospital, antibiotic therapy, location of infection (URTI, LRTI), diagnostic methods and presence of co-infections.

The disease severity score used in this study is a modification of the severity score developed by Gern [13]. In our modified score, fever, cough, rhinorrhea and duration of illness >4 days count 1 point each. Apnea counts 3 points. Wheezing, cyanosis, retractions and tachypnea count 5 points each. The maximum score is 27.

### Statistical analysis

Demographic characteristics were extracted and frequencies were calculated using SPSS 18 (SPSS, Chicago, IL). The following three intimately linked analyses have been performed with the aim to find discriminative parameters for pertussis:

In the first analysis we attempted to uncover discriminative parameters for clinical suspicion of pertussis by comparing clinical suspicious RT-PCR-positive cases (group A and C, n = 9) versus clinical suspicious PCR-negative cases (group B and D, n = 62) (analysis took place on both winter seasons). In the second analysis (restricted to the second season) we tested which clinical parameters distinguished clinical suspected pertussis cases (group C, n = 7) from non-suspected pertussis cases (group E, n = 7) cases. In the third analysis (restricted to the second season): all RT-PCR-positive samples (group C and E, n = 14) were compared to all RT-PCR negative samples (group D and F, n = 292) to further analyze predictive clinical parameters.

To test differences in (multiple) clinical parameters of two groups multivariate analysis were performed using a non-parametric test called Permanova (for details see Appendix) [14]. If the multivariate analysis showed significant differences between the groups tested, post-hoc univariate analyses were performed using a Kruskall Wallis and binomial tests (details see Appendix) to see which parameters contributed to this difference. The false discovery ratio was used to control for the family wise error rate [15]. To visualize the (multidimensional) differences between the groups, a non-metric dimensional scaling (NMDS) was applied, which displays the patients according to their similarity to each other [16] (for details see Appendix). Analysis were performed using the statistical software ‘R’ (R Development Core Team [17]). Significance was determined at a level of α < 0.05.

### Medical ethical approvement

The Evaluation of Viral Diagnostics on Respiratory Infections in Children trial protocol was approved by the regional medical ethics committee and The Central Committee on Research Involving Human Subjects (known by its Dutch initials, CCMO, Centrale Commissie Mensgebonden Onderzoek) number NL13839.098.06.

## Results

### Patient enrollment

In total, 776 NWSs were analyzed. We excluded 163 because they were obtained from children who did not meet inclusion criteria for various reasons (see Figure 1). Of the 613 samples who did met the inclusion criteria, we excluded another 31 samples because the clinical data were incomplete. Of the remaining 582 NWSs (from 542 patients), 209 were included in the first season and 373 cases in the second season. Both seasons included in our study were seasons with high incidence of pertussis in the Netherlands (40–50 cases per 100.000) [18].

Seventy-one samples were taken from children with clinical suspicion for pertussis (1^st^ season n = 22/2^nd^ season n = 49). PCR analysis on pertussis was performed on these samples prospectively. In the second season 324 samples from non-suspicious children were retrospectively analyzed. Unfortunately, of 67 samples (out of 324 NWSs) too little material was left for RT-PCR after thawing, leaving 257 samples for retrospective analysis. A summary of in- and exclusion numbers is given in Figure 1. Mean age was 8,2 months (median 4.2) and 57,7% was male. URTI was diagnosed in 324/582 (55,7%) children. Exclusively LRTI in 2/582 (0,3%) and combined infections in 232/582 (39,9%) children. Paroxysmal cough was found in 97/582 (16,7%) children and 434/582 (74,6%) were admitted to the pediatric ward.

### Outcomes

Table 1 shows the number of pertussis cases diagnosed in each of the two seasons. In the first winter season clinical suspicion of pertussis was raised in 22 cases (group A and B) out of 209 (10,5%), two cases of pertussis were diagnosed by RT-PCR (2/22 = 9,1%). In the second winter season clinical suspicion of pertussis was raised in 49 cases (group C and D) out of 373 (13,1%), seven cases of pertussis were diagnosed by RT-PCR (7/49 = 14,3%) (group C). In the second season another seven cases were found in the non-suspicion group (7/257 = 2,7%) (group E). All NWSs were also tested for *B. parapertussis*, but no cases were found in our study.

**Table 1:** **Number of pertussis cases per season**

	First winter	Second winter
	Season 2008–2009 (n = 209 )	Season 2009–2010 (n = 373 )
**Clinical suspicion of pertussis**		
Clinical suspicion of pertussis (%)	22/209 (10,5)	49/373 (13,1)
Prospective PCR *B, pertussis* positive n/N (%)	2/22 (9,1)	7/49 (14,3)
**No clinical suspicion of pertussis**		
No clinical suspicion of pertussis (%)	187/209(89,5)	324/373 (86,9)
Material for retrospective PCR	0/187 (0,0)	257/324 (79,3)
Retrospective PCR *B. pertussis* positive, n/N (%)	*NA*	7/257 (2,7)
***Total number of Pertussis cases, n/N (%)***	*2/209 (1,0)*	*14/373 (3,8)*

NA: not applicable.

The clinical features of the four identified groups in the second winter are shown in Table 2. Fourteen cases tested positive for pertussis (group C, (clinical suspicion RT-PCR positive), and group E (non-suspicion RT-PCR positive)). All fourteen pertussis patients showed signs of an URTI, but that symptom was one of the study inclusion criteria. Ten out of these 14 were also diagnosed with LRTI. All four patients with exclusively URTI fell into the clinical non-suspicion group. Only one patient in the non-suspicion group suffered from paroxysmal cough. Viral co-infections were found in 11 out of 14 cases: in the suspected pertussis group 6 out of 7 had viral co-infection (1 virus detected n = 6) in the unsuspected pertussis group 5 cases had viral co-infection (1 virus detected n = 3; 2 viruses detected n = 2). Influenza A, RSV B, Rhino- and Bocavirus were the most common pathogens found. All children in the clinical suspicion group were prescribed claritromycin, in case of suspected pneumonia amoxicillin was prescribed as well. Of the pertussis cases, 6/14 were appropriately immunized, 4 were too young to have received completed immunization and 4 were not immunized based on their parental beliefs. Of the 14 cases 11 were admitted to the pediatric ward: 6/7 (86%) in the suspected pertussis group and 5/7 (71%) in the unsuspected pertussis group.

**Table 2:** **Features of cases in the second season, divided in four groups**

	Clinical suspicion	Clinical suspicion	Non-suspicion	Non-suspicion
	RT-PCR positive	RT-PCR negative	RT-PCR positive	RT -PCR negative
	N = 7	N = 42	N = 7	N = 250
	(Figure 1, group C)	(Figure 1, group D)	(Figure 1, group E)	(Figure 1, group F)
Male, n	4	30	1	147
Age, months, mean (range)	14,8 (1,2-49,6)	4,8 (0,1-21,7)	3,8 (0,6 - 8,7)	7,6 (0,1-89,4)
Clinical features				
In-hospital cases, n	6 (85,7%)	30 (71,4%)	5 (71,4%)	182 (72,8%)
Fever, n	3 (42,9%)	18 (42,9%)	1 (14,2%)	131 (52,4%)
Coughing, n	7 (100%)	41 (97,6%)	7 (100%)	207 (82,8%)
Rhinorrhea, n	7 (100%)	39 (92,9%)	7 (100%)	231 (92,4%)
Paroxysmal cough, n	6 (85,7%)	25 (59,5)	1 (14,2%)	27 (10,8%)
CRP > 40 mg/L, n	0 (0%)	3 (7,1%)	1 (14,2%)	26 (10,4%)
Oxygen therapy necessary, n	2 (28,6%)	15 (35,7%)	2 (28,6%)	105 (42,0 %)
Nasogastric feeding, n	1 (14,3%)	5 (11,9%)	0 (0,0%)	27 (10,8%)
Wheezing, n	3 (42,9%)	15 (35,7%)	1 (14,2%)	113 (45,2%)
Apnoe, n	0 (0%)	3 (7,1%)	0 (0,0%)	10 (4,0%)
Days of illness, mean (range)	18,3 (5–37)	9,7 (2–42)	10,4 (5–18)	8,3 (1–35)
Disease severity score, mean (range)	9,9 (0–19)	11,7 (0–24)	9,6 (3–18)	13,4 (0–27)
Disease severity score				
<6	2	9	2	65
7-13	3	16	3	55
14-19	2	14	2	75
>20	0	3	0	55
Clinical diagnosis				
Exclusively URTI	0	23	4	133
Exclusively LRTI	0	0	0	1
Combined URTI/LRTI	7	18	3	106
No URTI or LRTI	0	1	0	10
Pertussis diagnosis				
PCR throat swab material, n	4	x	0	x
PCR NWS, n	3	x	7	x
Serology, positive, n	1	x	0	x
Culture, positive n	0	x	0	x
Viral (co)infection				
None	1	7	2	37
1 virus	6	21	3	146
≥ 2 virusses	0	14	2	67
Antibiotic therapy				
None	0	26	4	158
Amoxicillin, n	1	9	2	94
Claritromycine, n	7	7	1	8

**Figure 2:**
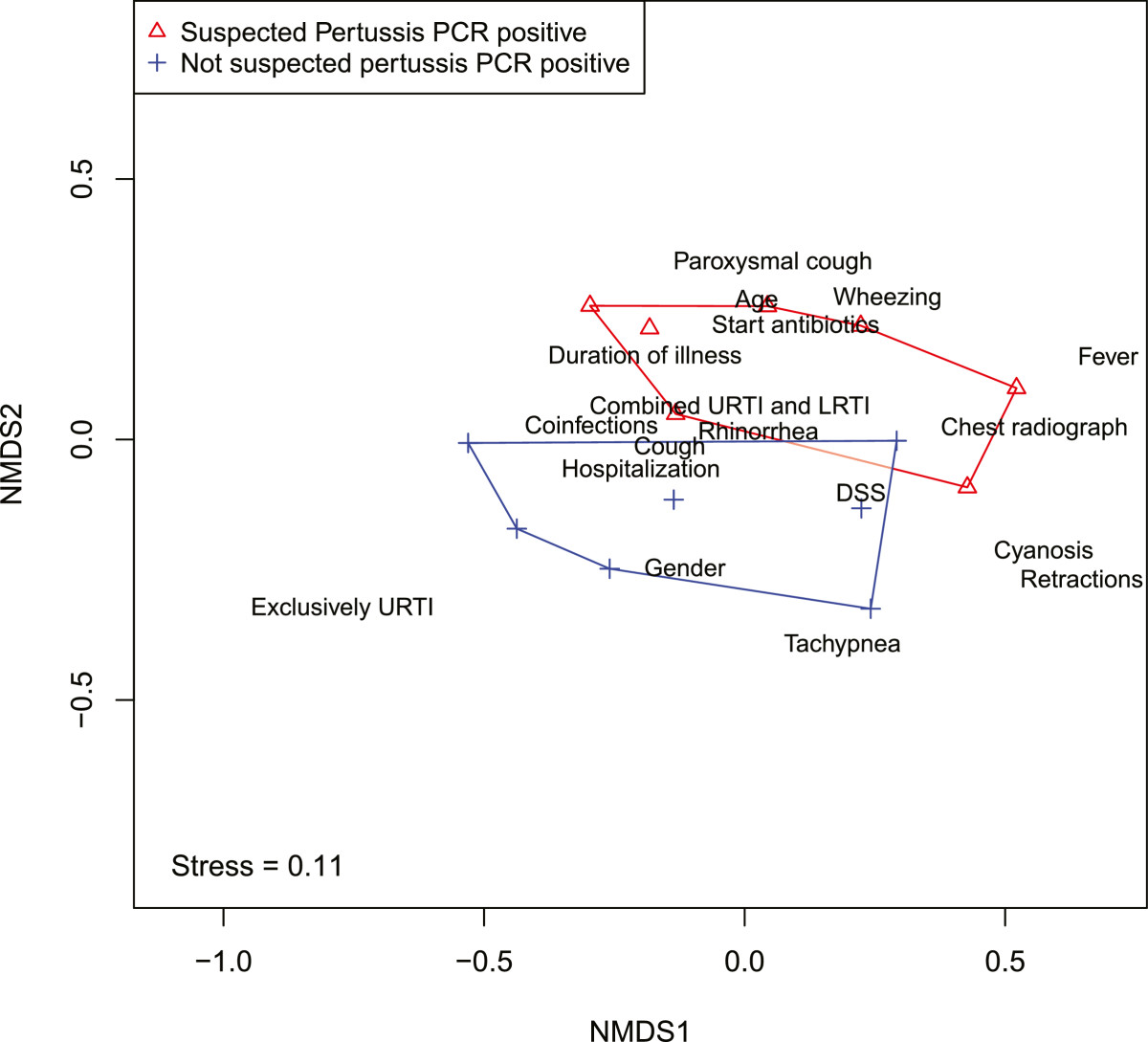
**Visualization of the differences (in characteristics) between the suspected and unsuspected pertussis group.**

Multivariate analyses were performed on the groups described in analysis 1, 2 and 3. No significant differences were found between the clinical suspicion RT-PCR positive group (group A and C) and the clinical suspicion RT-PCR negative group (group B and D) (Analysis 1: p-value 0,12).Among those who tested positive for pertussis in the second season (group C and E), multivariate analysis showed significant differences between the suspected (group C) and the unsuspected pertussis group (group E) (Analysis 2 p = 0,03). Figure 2 is a visualization of the differences (in characteristics) between both groups and shows that the two groups can be separated from one another in space, which means that the two groups differ in clinical parameters.

To test which factors contributed to the difference between both groups, a post-hoc test was performed, in which each factor was analyzed separately (results shown in Table 3). In the unsuspected pertussis group (group E) less children had paroxysmal cough (p < 0,01) and less antibiotics were prescribed (p = 0,01). Location of infection also significantly differed between the two groups: more URTIs were found in the unsuspected pertussis group (p = 0,01) and more combined URTI and LRTI were found in de clinical suspected group (p = 0,01) (group C). Other clinical findings (feeding support, presence of fever) did not differ between the two groups. Considering that ARI was the main characteristic for inclusion, general symptoms belonging to ARI (cough, rhinorrhoea) were present in all participants and could therefore not be included in this post-hoc test. Apnea was not observed in one of the groups, therefore this parameter was not included in the post hoc test as well.

**Table 3:** **Post-hoc univariate analysis (analysis 2): PCR Pertussis positive: suspected (group III) versus non-suspected pertussis group (group V)**

Variable	P value
Age (months)	0,180^a^
Gender	0,112^b^
Coinfections	0,708^a^
Hospitalization	0,513^b^
Duration of Illness	0,140^a^
Fever	0,264^b^
Cough	Np
Rhinorrhea	Np
Apnea	Np
Wheezing	0,271^b^
Cyanosis	0,791^b^
Retractions	0,515^b^
Tachypnea	0,507^b^
Paroxysmal coughing	**0,0076** ^**b**^
Disease severity score	0,844^a^
Combined URTI and LRTI	**0,0132** ^**b**^
Exclusively URTI	**0,0136** ^**b**^
Chest radiograph	0,509^b^
Start antibiotics	**0,0122** ^**b**^

^a^Kruskal Wallis test for continuous variables.

^b^Binomial test for discrete variables.

np = not possible.

Significant differences noted as bold.

No significant differences were found between all RT-PCR positive cases (group C and E) and all RT-PCR negative cases (group D and F) (Analysis 3: p-value 0,48).

Since paroxysmal cough is considered one of the main symptoms of pertussis, we retrospectively assessed the frequency of paroxysmal cough in ARI. Paroxysmal cough was reported by the parents or observed by the clinician. Paroxysmal cough was often seen in children with ARIs (97/582 = 16,7%). In 43 of 97 cases with paroxysmal cough the consulting pediatrician had clinical suspicion of pertussis and decided to perform diagnostics, 8 out of these 97 were proven pertussis. In 54 of 97 cases with paroxysmal cough the consulting pediatrician had no clinical suspicion of pertussis. Frequently alternative diagnoses were noted in the medical chart, mostly bronchiolitis. Two-thirds of these cases were RSV positive, some were positive for other viruses. Retrospectively, only one positive pertussis case was found in this group.

## Discussion

In literature, paroxysmal cough is considered the most important classical symptom of pertussis; it is also a major criterion in clinical case definitions of de World Health Organization and Centers for Disease Control and Prevention. Also in the clinical case definitions proposed by the Global Pertussis Initiative Roundtable Meeting in 2011, paroxysmal cough is still considered a major criteria in children above 3 months of age [11]. However this study shows that clinical suspicion based on paroxysmal coughing only predicts accurately in about 10-15% of cases. Based on the second study season, theoretically 4 out of 5 pertussis cases were accurately recognized (100 – ((2,7/14,3) × 100) = 81,1%) and 1 out of 5 pertussis cases was missed ((2,7/14,3) × 100 = 18,9%) if pertussis diagnostics are only performed when the doctor has clinical suspicion of pertussis. In accordance with other reports [4, 8, 19], we observed that paroxysmal cough is not specific for pertussis. In the unsuspected pertussis group less children suffered from paroxysmal cough and more children had only symptoms of an URTI. This suggests that the current clinical case definitions of pertussis are not sufficient for diagnosing all pertussis cases. Especially the atypical and mild infections, more frequently seen in unvaccinated children, are missed. Moreover, these children are also at risk for developing severe life threatening apneas if treatment is delayed. We showed that paroxysmal cough is also seen in children with common viral respiratory infections, which implies that further research should focus on determining accurate clinical predictors of pertussis.

Our study suggests that reported incidences of pertussis underestimate the true incidences. Our study results are supported by seroprevalence studies that have shown that only 20–25% patients with positive serology recall symptoms during the preceding year [1, 20].

Different studies showed that in respiratory infections often more than one pathogen is found. It is not clear which pathogen is the primary causative agent and which associations between pathogens contribute to disease severity [21]. Also in pertussis, co-infections with respiratory pathogens often occur [9, 22]. In our study 11/14 (78,6%) patients with pertussis had viral co-infections. Co-infections did not seem to explain the differences found between the unsuspected and suspected pertussis group. We could not determine if these infections occurred simultaneously or consecutively. Clinical features of cases with mixed infections do not differ from those with only one organism present [4, 8, 22]. In cases of mixed infections, it is unclear which pathogen contributes most to the disease symptoms. It is possible that only one organism causes symptoms while the other micro-organism is only residing in the respiratory tract. Although some studies [23] suggest asymptomatic carriage of *B. pertussis*, situations in which *B. pertussis* is detected more likely reflect asymptomatic or mild infections. The much higher seroprevalence compared to the number of reported cases supports the idea of asymptomatic infections or carriage [1]. Additionally, in the situation of co-infection, coughing due to viral infection might contribute to transmission of pertussis as well.

The clinical severity of pertussis infections may be modified by vaccination. Fully vaccinated children are thought to have more silent or mild pertussis infections and more severe presentations are seen in unvaccinated children, especially at a young age [2, 5]. In the Netherlands children are vaccinated against pertussis with an acellular vaccine at the age of 2, 3, 4 and 11 months and 4 years [18]. In the study period (2007–2009), overall vaccination coverage for pertussis in the Netherlands was around 95% for infants, and 91% for toddlers. In both participating hospitals vaccine coverage was equal to the national percentage [18]. In our study 8 out of 14 children with pertussis were not immunized (yet), from which 4 cases were found based on clinical suspicion. Of these 8 children, five were less than two months of age and therefore not (yet) immunized. The other three children were not immunized for religious reasons. The other six children with pertussis were immunized with the acellular vaccine, one child was 4 years and fully immunized. The age of the other five varied between 3 and 9 months (mean 6.3 months). Vaccination strategy and type of vaccination are matter of interest in literature, duration of immunity might differ between de whole cell and the acellular vaccines [24]. In our study, almost all children were vaccinated with the acellular vaccine, since all children in The Netherlands born after January 2005 have been vaccinated with this type of vaccine. De Greeff et al. demonstrated that household contacts play and important role in transmission of pertussis to children and show that 1–3 years after vaccination children are again susceptible for pertussis [6]. It has also been shown that infection may occur shortly after vaccination, not always with typical clinical symptoms [3, 25] and depending on the efficacy of the vaccine used.

In this study we used RT-PCR to detect *B. pertussis,* mostly on NWS/nasopharyngeal aspirates and only a few times on throat swabs. The method of specimen collection depended on the experience in the local hospital. NWS/nasopharyngeal aspirates and throat swabs provide mucus that contains columnar respiratory epithelial cells, the target cell for attachment of *B. pertussis* [2]. In 2005, the Pertussis Consensus Group recommended NWSs/nasopharyngeal aspirates as the optimal sample for RT-PCR in infants and throat swabs as an possible alternative in older children [26]. In some studies throat swabs are considered suboptimal to nasopharyngeal swabs, but Holberg et al. showed that they have similar sensitivity [27]. In our study, PCR on throat swabs was done in a minority of cases (11 of which four tested positive for pertussis). RT-PCR is a rapid, sensitive and specific diagnostic test, may detect infection early and later in disease progression and may be used in vaccinated children, in whom serology is unreliable [2, 7]. It is suggested that RT-PCR for pertussis remains positive at least three weeks from onset of catarrhal symptoms [2]. After this period, the usefulness of RT-PCR declines and serology becomes more important in unimmunized children [7].

The PCR target used for diagnosis in this study was IS481, Consequently, we cannot distinguish between *B. pertussis, B. holmesii* and *B. bronchiseptica. B. holmesii* has been found in three Dutch patients with pertussis-like symptoms [28] but the prevalence appears to be low [29]. Furthermore, B. holmesii has mainly been found in adolescents and adults [30]. Therefore, we consider it is unlikely that our data are an overestimation of true data. The use of the IS481 target instead of species-specific targets in a clinical setting is further supported by data from a recent study of Spicer et al. He reported that 222 out of 520 (42.7%) IS481 positive nasopharyngeal specimens were unable to be confirmed as having *B. pertussis* or *B. holmesii* by species specific PCR assays. This likely reflects the greater sensitivity of the IS481 target, because there are more targets per organism than in an assay using species specific targets [31].

There are limitations to this study. First, the number of RT-PCR-positive pertussis samples is small, limiting the application of the results to the general population. Second, serology was not performed. Had serology been included, more pertussis cases might have been found. Another limitation was that no retrospective data on the first season were available. Also, we cannot exclude some selection bias in our study, since most children were referred to the hospital only after initial assessment by a primary physician, as is common in the Dutch healthcare system. Therefore, patients with milder disease might be underrepresented. Furthermore it might be important to evaluate what makes a physician think whether a patient has pertussis, considering the fact that our study and other studies show that paroxysmal cough is not a specific predictor of pertussis [4, 8, 19]. Also, the possible differing interpretations of the definition of paroxysmal cough between physicians could have led to interpretation bias, with both the risk of over- and underestimation of clinical suspect pertussis cases.

We believe that the results of our study adequately reflect the situation in national hospitals and most likely also internationally: in children with respiratory infections, pertussis diagnostics are not routinely performed, but only in case of clinical suspicion. The current clinical criteria for pertussis are all based on paroxysmal cough, which, as we showed, is not a good predictor in atypical pertussis infections. The study population of previously healthy children with ARI are comparable to other pediatric populations. Therefore we believe our results are also generalizable to similar settings.

## Conclusions

Children with pertussis may present with classic or atypical symptoms. Presentation may mimic a viral respiratory tract infection, with the consequence of continuous spreading of pertussis in the population. Our study showed that when the initiation of pertussis diagnostics is based on clinical suspicion, about 1 in 5 cases (19%) is missed. Despite being widely accepted as a clinical criteria on pertussis, paroxysmal cough is not a good predictor of a pertussis infection in our study. In our opinion pertussis cannot be diagnosed solely on clinical grounds. Therefore, since pertussis is a treatable disease, we stress the importance of *B. pertussis* diagnostics, especially in children with respiratory symptoms. We advocate that if one searches for a causative pathogen, one should search for treatable pathogens like *B. pertussis*. Although more frequent laboratory diagnostics may help to limit spread of pertussis in the population, it comes with high costs. Studies investigating the cost-effectiveness are warranted.

## Appendix

### Permanova

PERMANOVA is the non-parametric analogue of the MANOVA [14]. A permanova can take both continuous as discrete variables to calculate significant differences between multiple groups. It does so by testing whether the similarity of the individuals, based on their characteristics, within a group is smaller than the similarity of individuals from different groups. In this study Gower distance was taken a distance measure, since this measure can handle a mix of datatypes (binary, discrete and continuous). To test whether the distances within a group are smaller than between groups a permutation test was performed, by randomly assigning group labels 1) Clinical suspicion PCR positive (n=9) vs Clinical suspicion PCR negative (n=62); 2) Clinical suspicion PCR positive (n=7) and no clinical suspicion PCR positive (n=7); and 3) all PCR positive (14 patients) vs. all PCR negative (292) to each patient. This randomization was repeated a large number times (999) and gives a null distribution of distance values (hypothesis: no differences between groups). Subsequently, the F test was used to determine if the observed outcome significantly differed from the null expectation. The larger the value of F, the more likely it is that the null hypothesis is wrong, and that the observed outcome is not likely to have occurred by chance [14]. Like a MANOVA, a permanova is sensitive to differences in variance among groups, particularly when sample sizes differ substantially. Since we cannot apply a permanova on the full group sizes, analysis 1 and 3 were performed slightly differently. For analysis 1, we randomly took 9 patients from the group of 62 and compared those with the suspected PCR positive. We then tested for homogeneity of variances (using the method described by Anderson [32]) and run the permanova as described above for group 2. This procedure was repeated 1000 times. The two groups can be considered significantly different from each other when more than 950 permanova’s (out of 1000) show a significant difference between the groups [33]. This procedure was also used for comparing the two groups in analysis 3. Both analysis 1 and 3 showed no significant differences between the groups. Thus there are no significant differences between the clinical suspicion PCR positive and clinical suspicion PCR negative group. Also no differences were found between all PCR positive versus all PCR negative cases.

### Binomial test

Given the significant differences between the clinical suspected and unsuspected group, we subsequently tested which variables contributed to the differences among the groups (post-hoc test). For the binary variables, we used a binomial test. We tested the probability that the frequency of the symptoms being present in the different groups could be due to chance. To do so we compared the ratio of frequencies of a particular symptom in two groups to the ratio of frequencies of the two groups assuming no differences between the two groups (null hypothesis: each patient, regardless which group it is in, has an equal probability to show a particular symptom). The frequency under the null model was generated by drawing 7 and 7 times from a binomial distribution with probability of 0.5. This was repeated 10,000 times. The thus derived frequency distribution was compared to the observed frequency. If the observed frequency falls outside the 95% confidence interval of the null distribution, we consider it unlikely that the observed frequency distribution is due to chance.

### Non metric dimensional scaling (NMDS)

The difference between the patients of the suspected pertussis and unsuspected pertussis group was tested using a PERMANOVA. The test for significant differences in the PERMANOVA is based on the dissimilarity matrix and tests whether patients in a group are more similar to each other (within a group) than between groups. Recall that the dissimilarity matrix is calculated based on the characteristics of the patients and summarizes how similar patients are, based on their characteristics, in one number. Non-metric multidimensional scaling (NMDS) is a way to visualize these dissimilarities in a chosen number of dimensions (in this paper two). The position in two dimensions of the individual patients is shown such that the rank order of the distance between the patients in the plot agrees with the dissimilarities of the patients in dissimilarity matrix. The degree to which the rank order distances agree with the dissimilarities is called “stress”. The lower the stress the better. The NDMS procedure seeks the ordination with lowest stress.

## Authors’ original submitted files for images

Below are the links to the authors’ original submitted files for images. Authors’ original file for figure 1 Authors’ original file for figure 2

## Abbreviations

ARI: Acute respiratory infection
*Bp*: *Bordetella pertussis*
CRP: Complement-reactive protein
hMPV: Human metapneumovirus
LRTI: Lower respiratory tract infection
NWS: Nasal wash specimen
PCR: Polymerase chain reaction
RSV: Respiratory syncytial virus
RT-PCR: Real-time polymerase chain reaction
URTI: Upper respiratory tract infection.
