# Supplementary figures and images for: Bordetella pertussis: an underreported pathogen in pediatric respiratory infections, a prospective cohort study
Source: BMC Infect Dis. 2014 Sep 30;14:526. doi: 10.1186/1471-2334-14-526 (PMC4261543; doi:10.1186/1471-2334-14-526)

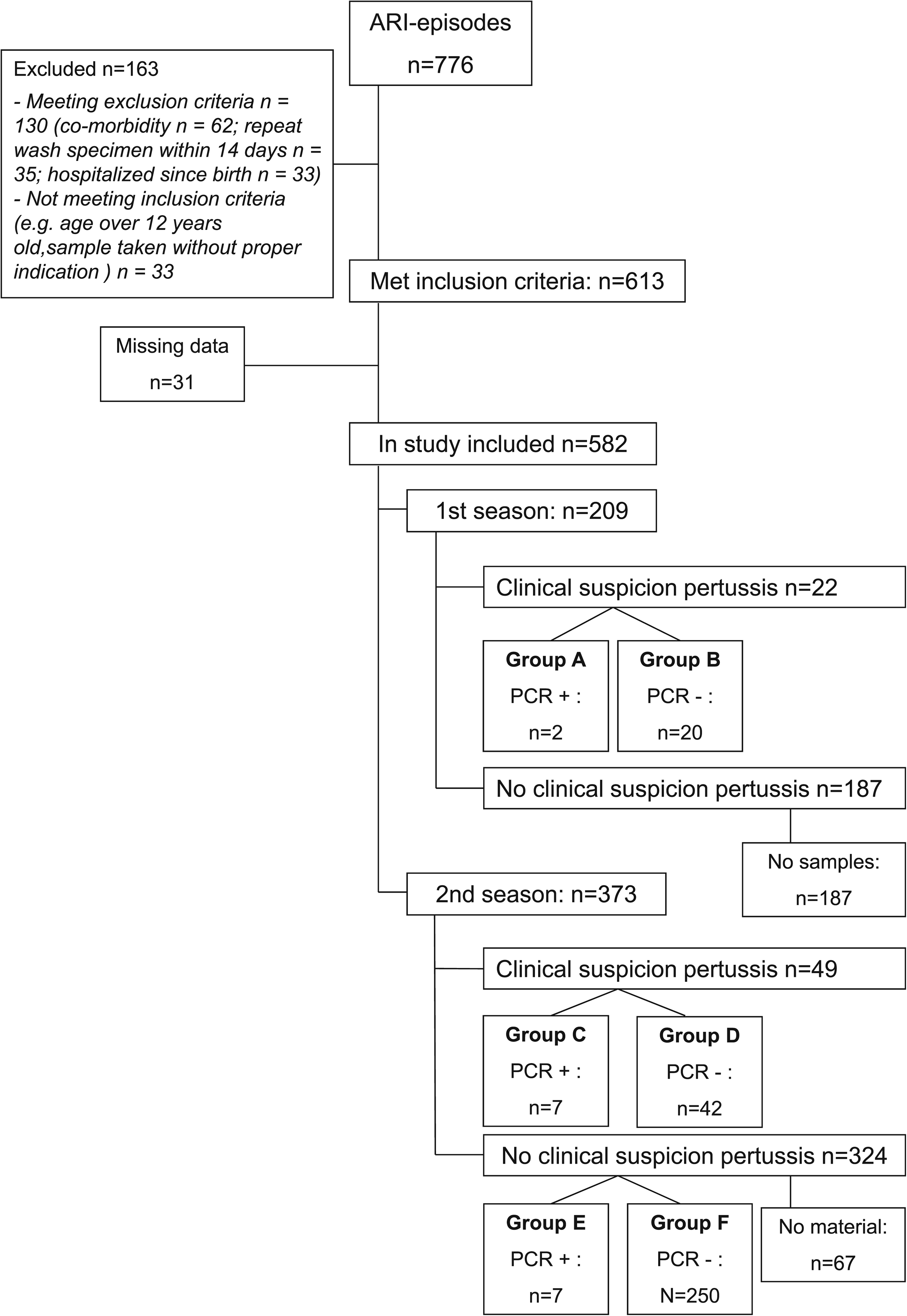

Supplement: Supplementary file 1 — Authors’ original file for figure 1 [file 12879_2014_3847_MOESM1_ESM.tif]

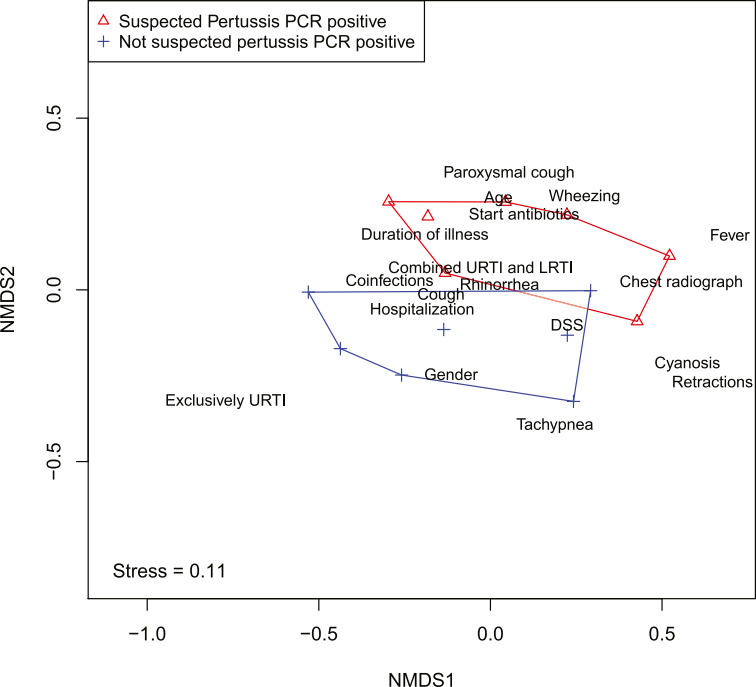

Supplement: Supplementary file 2 — Authors’ original file for figure 2 [file 12879_2014_3847_MOESM2_ESM.pdf]
